# Positive association between *ATP2B1* rs17249754 and essential hypertension: a case-control study in Burkina Faso, West Africa

**DOI:** 10.1186/s12872-019-1136-x

**Published:** 2019-06-26

**Authors:** Herman Karim Sombié, Jonas Koudougou Kologo, Daméhan Tchelougou, Serge Yannick Ouédraogo, Abdoul Karim Ouattara, Tegwindé Rebecca Compaoré, Bolni Marius Nagalo, Abel Pegdwendé Sorgho, Issoufou Nagabila, Serge Théophile Soubeïga, Florencia Wendkuuni Djigma, Albert Théophane Yonli, Patrice Zabsonré, Hassanata Millogo, Jacques Simporé

**Affiliations:** 1 Department of Microbiology and Biochemistry, Laboratory of Molecular Biology and Genetics (LABIOGENE), University Ouaga I Prof. Joseph Ki-Zerbo, P.O. Box 7021, Ouagadougou 03, Burkina Faso; 2 Saint Camille hospital in Ouagadougou (HOSCO), Burkina Faso, P.O. Box 444, Ouagadougou 01, Burkina Faso; 3 Pietro Annigoni Biomolecular Research Center (CERBA), P.O. Box 364, Ouagadougou 01, Burkina Faso; 4 Faculty of Medicine, University Saint Thomas d’Aquin, P.O. Box 10212, Ouagadougou, Burkina Faso; 5 University Hospital Center-Yalgado Ouedraogo (CHUYO), Ouagadougou, Burkina Faso

**Keywords:** *ATP2B1*, *STK39*, Essential hypertension, Burkina Faso

## Abstract

**Background:**

Genetic and environment play a significant role in the etiology of essential hypertension (EH). Recently *STK39* rs3754777, *ATP2B1* rs2681472 and rs17249754 have been associated with BP variation and hypertension. In this study we aimed to determine firstly whether index variants were associated with the risk of developing EH in Burkina Faso and secondly to characterize cardiovascular risk markers.

**Methods:**

We conducted a case-control study with 380 participants including 180 case subjects with EH and 200 control subjects with normal BP. We used TaqMan genotyping assays with probes from Applied Biosystems to genotype polymorphisms using the 7500 Real-Time PCR System. Biochemical parameters were measured using chemistry analyzer COBAS C311.

**Results:**

T-test showed that cardiovascular risk markers such as body mass index, waist circumference, blood sugar, total cholesterol and triglycerides were significantly higher in hypertensive compared to normotensive (all *p* <  0.05). Binary logistic regression analysis revealed in decreasing order that overweight, family history of hypertension, central obesity and alcohol intake increased the risk of developing EH (all OR > 3.8; all *p* <  0.001).

In genetic level we observed that individuals carrying the AA+AG genotype of *ATP2B1* rs17249754 had a low risk of developing EH than those carrying the GG genotype (OR = 0.48 [95% CI: 0.31–0.75] *p* = 0.001) and the A allele frequency in the cases was significantly lower than that of the controls (OR = 0.56 [95% CI: 0.38–0.82] *p* = 0.003). We also observed that *ATP2B1* rs17249754 was significantly associated with higher SBP and DPB in case and control groups (GG versus AG + AA; *p* <  0.05), *ATP2B1* rs2681472 was significantly associated with higher SBP only in case and control group (AA versus AG + GG; *p* <  0.05), *STK39* rs3754777 was not significantly associated with any of the BP traits (CC versus CT + TT; *p* > 0.05).

**Conclusion:**

Our results confirmed the significant association of *ATP2B1* rs17249754 with the risk of developing EH in Burkinabe and showed an increase of cardiovascular risk markers levels in subjects with EH.

**Electronic supplementary material:**

The online version of this article (10.1186/s12872-019-1136-x) contains supplementary material, which is available to authorized users.

## Background

Cardiovascular diseases are the leading cause of mortality in the world and represent 31% of global deaths [1]. The common risk factors for developing cardiovascular disorders are obesity, arterial hypertension, diabetes and dyslipidemia [2].

Hypertension is the main leading causes for morbidity and mortality of cardiovascular diseases and affects about one-third of adults worldwide each year [3, 4]. Hypertension was once considered rare in Africa, but currently it has become a public health concern. Several studies estimated at 16.2% the overall prevalence of hypertension in 74.4 millions of hypertensive individuals in sub-Saharan Africa and the number of affected individuals will increase by 68% (125.5 million) by 2025 [5]. The causes of EH which accounts for 95% of cases of hypertension remain largely unknown, however interplay among genetic and non genetic factors might contribute to its etiology [6]. An estimated 30–60% of blood pressure variation is explained by genetic factors [7]. Determinants also include life-style [8], obesity [9] and environment which can impact blood pressure or risk of hypertension through the influence on gene expression or through interaction with gene products [10].

Recently, with the enormous progress made in molecular Biology domain, Genome-wide association studies (GWAS) have identified news genes and their variants which are associated with blood pressure variations and the risk of hypertension such as *STK39* rs3754777 [11], *ATP2B1* rs2681472 and rs17249754 [12]. In addition, other reports confirmed these associations in Asian and European population [7, 13–16], but not all.

In this work, we genotyped index variants from these 2 candidate loci identified by studies and examined for the first time the association between them and systolic blood pressure (SBP), diastolic blood pressure (DBP) and the risk of developing EH in Burkina Faso, West Africa and in a second time we characterized some cardiovascular risk markers in patients with EH. The results will be instrumental in the future for a better clinical management of cardiovascular diseases in the country.

## Methods

### Study design

This case-control study was performed in Burkina Faso located in West Africa. A detailed description of our study population has been published previously [17]. Briefly 380 age-sex matched subjects from 20 to 75 years were recruited in the same geographical area of central region in Burkina Faso, including 180 subjects newly diagnosed with EH as case group and 200 subjects having normal blood pressure as control group.

Patients with EH were diagnosed by the cardiologist in the absence of secondary causes and recruited from the service of cardiology of Saint Camille hospital and the University Hospital Center Yalgado Ouedraogo of Ouagadougou. Hypertension was defined as systolic blood pressure (SBP) ≥ 140 mmHg and/or diastolic blood pressure (DBP) ≥ 90 mmHg [18].

Controls were subjects with SBP < 130 mmHg and DBP < 80 mmHg without antihypertensive treatments (to avoid pre-hypertension) and without any previous history of high blood pressure. They were recruited in general consultation in the same centers.

Patients who are already taking antihypertensive medications, patients with secondary hypertension or chronic diseases and pregnant women were excluded to avoid confusion.

### Samples and data collection

We recorded using a questionnaire followed by a medical examination, socio-anthropometric parameters (age, sex, waist circumference, weight and height), lifestyle (smoking, alcohol intake), family history of HTA and clinical parameters such as systolic blood pressure (SBP) and diastolic blood pressure (DBP).

Information about the participant’s age (years) was based on their self-reported birth year.

Body weight and height were measured respectively by using standardized scale and stadiometer. Body mass index (BMI) was obtained by dividing a person’s weight (kilograms) by the square of the person’s height (meters). Overweight was defined when BMI ≥ 25 Kg/m^2^.

Waist circumference (WC) was a measure of the distance around the abdomen in centimeter while the subject was at minimal respiration by using measuring tape. Central obesity was determinate when WC > 102 cm for men and WC > 88 cm for women [19].

Smoking status and alcohol intake were dichotomized respectively into smokers versus nonsmokers and drinkers versus nondrinkers.

We defined family history of hypertension like having someone in your family (a blood relative such as a mother, father, sister, or brother) who has or had high blood pressure before the age of 60 years old.

Blood pressure values were measured 3 consecutive times, after 10 min of seated rest before the first measurement and 5 min intervals between each measurement with a manual aneroid sphygmomanometer. The average of the 3 measurements was used for analyses.

We also collected 8 ml of fasting venous blood in tube containing anticoagulant (EDTA) and tube without anticoagulant for analyses. Serum was used to determine High density lipoprotein cholesterol (HDL-c), low density lipoprotein cholesterol (LDL-c), total cholesterol (TC), triglycerides and blood sugar using the COBAS C311 chemistry analyzer (*Roche-Hitachi, France*).

### DNA extraction and genotyping of polymorphisms

Genomic DNA was isolated from peripheral blood white cells using the standard salt fractionation method as described by Miller *and al.* in 1988 [20]. The purity and concentration of DNA were assessed using a Biodrop μLITE (Isogen Life Science, N.V/S.A, Temse, Belgium).

Polymorphisms were genotyped by TaqMan allelic discrimination assays with probes labeled with the fluorophores FAM/VIC (C_16057071_10, C_34612217_10 and C_27474774_10) purchased from Applied Biosystems (ABI, Applera International Inc., Foster City, CA, USA). PCRs were performed according to the protocol established by the manufacturer in 25 μL reaction volume including 5 μL of DNA, 12.5 μL of TaqMan Universal PCR Master Mix, 6.25 μL of sterilized water and 1.25 μL of SNP mix (40X) using 7500 Fast real time PCR system. Fluorescence was analyzed with the 7500 FAST Sequence Detection Software version v.2.1 (Applied Biosystem).

### Statistical analysis

Statistical Package for Social Sciences (SPSS Version 20.0) and Epi Info (Version 6.0) were used for data analysis.

Sample size and power calculations were conducted (Epi Info Version 6.0) by using following values: Two-sided confidence level of 95%, power of 80%, ratio of controls to cases 1.1, the proportion exposed in the control group with 50%, odds ratio of 1.8 or greater.

Quantitative variables were expressed as mean ± standard deviation and comparison between groups was assessed with Student’s t-test. Pearson correlation test was used to established correlation.

Investigation of factors increasing the risk of developing EH were done with binary logistic regression analysis (forward stepwise method) by taking hypertensive status as a dependent variable and including some conventional cardiovascular risk markers such as gender (male/female), overweight (yes/no), central obesity (yes/no), alcohol consumption (yes/no), Smoking (yes/no) and family history of HTA (yes/no).

Associations between polymorphisms and EH and/or blood pressure were established by comparing genotypic and allelic frequencies between cases and controls using the chi-squared test and averages of arterial blood pressures between genotypes using Student’s t-test respectively.

For all analyses, difference was statistically significant when *p* <  0.05.

## Results

### Quantitative characteristics

The characteristics of the study population are given in Table 1. We included 180 subjects with EH as cases and 200 subjects with normal blood pressure as controls. Hypertensive patients were mostly female (58.68%). T-test demonstrated that means of BMI (MD = 4.89; *p* <  0.001), WC (MD = 10.69; *p* <  0.001), serum level of blood sugar (MD = 2.25; *p* <  0.001), total cholesterol (MD = 0.78; *p* = 0.001) and triglycerides (MD = 0.22; *p* = 0.03) were significantly higher in cases compared to controls.

**Table 1:** General Characteristics of the study population

Parameters	Total *n* = 380	Cases *n* = 180	controls *n* = 200	MD	CI (95%)	*p* values
Sex (M/F)	157/223	74/106	83/117	–	–	1
Age (years)	48.98 ± 11.32	48.21 ± 10.09	49.67 ± 12.31	−1.45	− 3.74 - 0.82	0.21
SBP (mmHg)	140.50 ± 30.36	167.58 ± 20.0	116.13 ± 11.6	51.45	48.18–54.71	< 0.001∗
DBP (mmHg)	84.95 ± 16.81	98.73 ± 12.15	72.55 ± 8.90	26.18	24.05–28.32	< 0.001∗
BMI (kg/m^2^)	25.90 ± 6.45	28.48 ± 7.18	23.58 ± 4.63	4.89	3.68–6.10	< 0.001∗
WC (cm)	88.87 ± 12.60	94.50 ± 13.33	83.81 ± 9.39	10.69	8.38–13.00	< 0.001∗
Blood sugar (mmol/l)	5.10 ± 1.57	5.94 ± 2.84	3.69 ± 1.66	2.25	1.36–3.14	< 0.001∗
HDL-c (mmol/l)	1.27 ± 0.52	1.38 ± 0.55	1.08 ± 0.42	0.29	0.04–0.46	0.002∗
LDL-c (mmol/l)	3.03 ± 1.14	3.17 ± 1.10	2.60 ± 1.15	0.57	0.17–0.96	0.005∗
TC (mmol/l)	5.00 ± 1.36	5.13 ± 1.21	4.34 ± 1.48	0.78	0.32–1.24	0.001∗
Triglyceride (mmol/l)	1.24 ± 0.68	1.23 ± 0.56	1.00 ± 0.65	0.22	0.01–0.43	0.03∗

Values are Median ± SD for continuous variables; Cases versus controls (t-test); ∗ Significant *p* value; *HDL-c* high density lipoprotein cholesterol, *LDL-c* low density lipoprotein cholesterol, *TC* total cholesterol, *M* male, *F* female, *BMI* body mass index, *WC* waist circumference, *DBP* diastolic blood pressure, *SBP* systolic blood pressure, *MD* means difference

We determined Pearson index and we showed positive correlation of age, BMI, WC, blood sugar with systolic and diastolic blood pressure in the study population (case + controls). Only BMI was positively correlated with all other variables studied such as serum level of blood sugar, TC, HDL-c, LDL-c and Triglycerides (data not shown). Additional file 1: Table S1.

Binary logistic regression analysis revealed that in decreasing order, overweight, family history of HTA, central obesity and alcohol intake increased the risk of developing EH (all OR > 3.87; all *p* <  0.001) Table 2*.*

**Table 2:** Factors increasing risk of essential hypertension

Factors	OR	95%CI	*p* values
Gender M/F	0.91	0.54–1.53	1
Overweight	5.24	2.92–9.43	< 0.0001∗
Central obesity	4.57	3.37–6.20	< 0.0001∗
Alcohol intake	3.87	2.47–6.08	< 0.0001∗
Smoking	1.37	0.66–2.8	0.46
Family history of HTA	5.19	3.32–8.13	< 0.0001∗

*WC* waist circumference, *BMI* body mass index, *M* male, *F* female, * significant *p* value

### Genetics analysis

Genotypic frequencies of the study polymorphisms are given in Table 3. A total of 380 subjects were genotyped for *ATP2B1* gene polymorphisms (rs2681472, rs17249754) and *STK39* gene polymorphism (rs3754777). All three polymorphisms genotypes didn’t deviate from the Hardy-Weinberg equilibrium (*p* > 0.05). In the general population, we found that genotypic frequencies of rs2681472 were 2.1% for GG, 16.1% for GA and 81.8% for AA; genotypic frequencies of rs17249754 were 2.6% for AA, 30.0% for AG, 67.4% for GG; genotypic frequencies of rs3754777 were 2.1% for TT, 25.3% for TC, 72.6% for CC.

**Table 3:** Distribution of the genotypes frequency in the study population

SNPs	Genotypes	Cases + Controls *n* = 380 (100%)	Cases *n* = 180 (100%)	Controls *n* = 200 (100%)
rs2681472	GG	8 (2.1)	4 (2.2)	4 (2.0)
	GA	61 (16.1)	27 (15.0)	34 (17.0)
	AA	311 (81.8)	149 (82.8)	162 (81.0)
HWE *p* value		0.09	0.24	0.30
rs17249754	AA	10 (2.6)	4 (2.2)	6 (3.0)
	AG	114 (30.0)	40 (22.2)	74 (37.0)
	GG	256 (67.4)	136 (75.6)	120 (60.0)
HWE *p* value		0.78	0.80	0.45
rs3754777	TT	8 (2.1)	4 (2.2)	4 (2.0)
	TC	96 (25.3)	46 (25.6)	50 (25.0)
	CC	276 (72.6)	130 (72.2)	146 (73.0)
HWE *p* value		0.85	1.00	1.00

*HWE* Hardy-Weinberg equilibrium

When we compared genotypic and allelic frequencies between cases and controls and average of SBP and DBP between genotypes, we observed that individuals carrying the AA+AG genotype of *ATP2B1* rs17249754 had a low risk of developing EH than those carrying the GG genotype (OR = 0.48 [95% CI: 0.31–0.75] *p* = 0.001). In addition, the A allele frequency of *ATP2B1* rs17249754 in the case group was significantly lower than that of the control group (allelic OR = 0.56 [95% CI: 0.38–0.82] allelic *p* = 0.003). We didn’t find any significant associations of *ATP2B1* rs2681472 and *STK39* rs3754777 with the risk of developing EH in our study population (all *p* > 0.05) Table 4.

**Table 4:** Associations between polymorphisms and risk of essential hypertension

SNPs		Cases, *n* (%) 180 (100%)	Controls, *n* (%) 200 (100%)	OR	CI (95%)	*p* values
rs2681472	GG versus AG + AA, *n* (%)	4 (2.2)	4 (2.0)	1.11	0.27–4.51	1.00
	GG + AG versus AA, *n* (%)	31 (17.2)	38 (19.0)	0.88	0.52–1.49	0.69
	A (%)	90.3	89.5	–	–	–
	G (%)	9.7	10.5	0.91	0.57–1.47	0.80
rs17249754	AA versus AG + GG, *n* (%)	4 (2.2)	6 (3.0)	0.73	0.20–2.64	0.75
	AA + AG versus GG, *n* (%)	44 (24.4)	80 (40.0)	0.48	0.31–0.75	0.001∗
	G (%)	86.7	78.5	–	–	–
	A (%)	13.3	21.5	0.56	0.38–0.82	0.003∗
rs3754777	TT versus TC + CC, *n* (%)	4 (2.0)	4 (2.0)	1.11	0.27–4.51	1.00
	TT + TC versus CC, *n* (%)	50 (28.0)	54 (27.0)	1.03	0.66–1.63	0.90
	C (%)	85.0	85.5	–	–	–
	T (%)	15.0	14.5	1.04	0.69–1.55	0.90

∗, significant *p value*; *NA* not available

We also observed that *ATP2B1* rs17249754 was significantly associated with higher SBP and DPB (GG versus AG + AA; *p* <  0.05) in cases and controls groups; *ATP2B1* rs2681472 was significantly associated with higher SBP only in case and control group (AA versus AG + GG; *p* <  0.05); *STK39* rs3754777 was not associated with any of the BP traits (CC versus CT + TT; *p* > 0.05). data not shown. Additional file 2: Table S2.

## Discussion

Hypertensive patients often present metabolic disorders which impact their quality of life and could increase their risk of developing cardiovascular diseases. In our study we characterized known cardiovascular risk markers in subjects with EH (cases) and normotensive individuals (controls). The results showed that subjects with EH had significantly higher levels of blood sugar (*p* <  0.001), BMI (*p* <  0.001) and total cholesterol (*p* <  0.05) compared to controls. These findings suggest that subjects with EH tend to develop disorders associated with metabolic syndrome as described previously [21]. These markers were positively correlated to the augmentation of systolic and diastolic blood pressure in our study population, supporting those several independent studies conducted in different population which showed that risk of hypertension increased with age [22] and BMI [23], and that dyslipidemia [24] and hyperglycemia [25] were more frequent in hypertensive compared to normotensive individuals. Epidemiological studies have demonstrated that Obesity and overweight were associated with an increase of cardiovascular risk markers [26], in our study we also found that BMI was positively correlated to all other cardiovascular risk markers such us SBP, DBP, HDL-c, LDL-c, TC, triglycerides and blood sugar level. Regular physical activities have been shown to reduce the risk of high blood pressure and could also help lipids repartition in the organism [27], in our study we also found that obese and overweight patients who are usually less active were the main group at risk of developing cardiovascular disorders as previously described [28].

In the genetic level we investigated the effect of genetic loci recently identified by GWAS with BP and the risk of developing EH. Our first gene of interest *ATP2B1* also named *PMCA1* (Plasma Membrane Calcium ATPase type 1) is located in chromosome 12, position 12q21.q23 and belongs to the P-type pump family [29]. It encoded a protein responsible for the regulated transport between the intracellular and the extracellular milieu of Ca^2+^, an ion which contributes to contraction-relaxation of vascular smooth muscles [30]. Mechanism by which *ATP2B1* gene influences blood pressure is not yet clear but, investigations showed its mRNA overexpression in hypertensive animal models compared to normotensive [31] and an excessive increase of blood pressure through vasoconstriction in *ATP2B1* gene deleted rats [32]. These findings may explain among other things the mechanism by which changes in the *ATP2B1* gene product levels are involved in BP regulation and risk of EH. In our study we observed that individuals carrying the AA+AG genotype of *ATP2B1* rs17249754 had a low risk of developing EH than those carrying the GG genotype (OR = 0.48; 95% CI = 0.31–0.75; *p* = 0.001). Furthermore, the A allele frequency of *ATP2B1* rs17249754 in the case group was significantly lower than that of the control group (allelic OR = 0.56; 95% CI = 0.38–0.82; allelic *p* = 0.003). The association of *ATP2B1* rs17249754 with hypertension has been previously shown in Koreans [12]. Subsequently GWA studies conducted by the Global Blood Pressure Genetics and CHARGE consortiums confirmed this association [13]. Recently Daily *and al.* in Korean, showed that carriers of the major allele G of *ATP2B1* rs17249754 were at greater risk of developing hypertension and that high Na intake and low Ca increased the risk more in major allele than among minor allele carrier, suggesting that people with the G allele can reduce risk of high blood pressure by having good calcium status [33]. Interaction of BMI, gender and *ATP2B1* rs17249754 in susceptibility to hypertension has been also reported in Han Chinese Population [15]. In our study, we were unable to perform sub-group analysis given the limited number of participants. Concerning *ATP2B1* rs2681472, it was found to be associated with hypertension firstly in 2009 by Levy *and al.* [7]. In our cohort, we didn’t find any significant association between it and essential hypertension, but we found that individuals carrying the AA genotype had high SBP than those carrying the GG + AG genotype. The association between rs17249754 and EHT demonstrate the potential role of *ATP2B1* in the regulation of blood pressure and treatment of EHT. Indeed, Okuyama and *al.*, showed that mice lacking *ATP2B1* had a higher response to CCBs for blood pressure-lowering effects than other anti-hypertensive drugs [34], and previously Tabara and *al.* showed that it may be a reduction in the expression of *ATP2B1* which leads to raised blood pressure in those with a *ATP2B1* risk allele for hypertension [35]. These data may suggest that hypertensive patients with variant G of rs17249754 (risk allele for hypertension) may also have a reduction in the expression of *ATP2B1* and therefore a better response to CCBs compared to other antihypertensive drugs. However, further studies are needed to confirm that.

Our second gene of interest *STK39*, encodes a serine-threonine kinase named STE20/SPS1-related proline/alanine-rich kinase (SPAK), which seems to impact blood pressure by its action on renal excretion of sodium through its interaction with the WNK kinase and co-transporters cation-chloride [36]. Experimental studies showed that rats in which SPAK and WNK interaction were blocked had a lower blood pressure [37]. In the present study, we didn’t find any significant association of *STK39* rs3754777 with EH as reported previously in Amish, non Amish [11], Belgian population [16] and male Han Chinese [38]. However, certain studies before ours reached to the same conclusion and failed to prove any association such as studies in British Caucasian [39] and Chinese Children [40].

In view of our results and other results obtained in previous studies, we can note a strong interaction between genetic variants and environmental and/or epigenetic factors, so that certain genetic variants only have significant effect in specific populations.

## Conclusion

In conclusion, our study confirmed the significant association between *ATP2B1* rs17249754 and EH in Burkinabe, suggesting the potential role of *ATP2B1* in the regulation of blood pressure. It also showed a significant increase of cardiovascular risk markers in individuals with EH compared to normotensive and once again invite clinicians to be looking at the level of BMI, WC and cholesterol in the management of EH. However, the present research has some limitations, particularly the small size of the study population. A large scale study will be necessary to fully comprehend the role of *ATP2B1* rs17249754 in the development of EH and response to CCBs in Burkina Faso.

## Additional files

Additional file 1: **Table S1.** Correlation between blood pressure and cardiovascular risk markers. This file shows correlation between known cardiovascular risk markers such as SBP, DBP, age, BMI, WC, serum level of blood sugar, TC, HDL-c, LDL-c and Triglycerides in the general study population. (DOCX 13 kb)

Additional file 2: **Table S2.** Distribution of systolic and diastolic blood pressure according to genotypes. This file presents the results of association analysis between genetic polymorphisms studied and SBP or DBP in cases and controls. (DOCX 13 kb)

## Data Availability

The datasets used and/or analyzed during the current study are available from the corresponding author on reasonable request.

## Abbreviations

*ATP2B1*: ATPase plasma membrane Ca^2+^ transporting 1
BMI: Body mass index
CERBA: Pietro Annigoni biomolecular research center
DBP: Diastolic blood pressure
EDTA: Ethylenediaminetetraacetic
EH: Essential hypertension
GWAS: Genome-wide association study
HDL-c: High-density lipoprotein cholesterol
LABIOGENE: Laboratory of molecular biology and genetics
LDL-c: Low-density lipoprotein cholesterol
MD: Means difference
PCR: Polymerase chain reaction
SBP: Systolic blood pressure
SD: Standard deviation
SPAK: STE20/SPS1-related proline/alanine-rich kinase
SPSS: Statistical package for the social sciences
*STK39*: Serine/threonine kinase 39
TC: Total cholesterol
WC: Waist circumference
WNK: With-no-K (Lys)
