# Supplementary material for: Positive association between ATP2B1 rs17249754 and essential hypertension: a case-control study in Burkina Faso, West Africa
Source: BMC Cardiovasc Disord. 2019 Jun 26;19:155. doi: 10.1186/s12872-019-1136-x (PMC6595568; doi:10.1186/s12872-019-1136-x)
Supplement: Supplementary file 1 — Table S1. Correlation between blood pressure and cardiovascular risk markers. This file shows correlation between known cardiovascular risk markers such as SBP, DBP, age, BMI, WC, serum level of blood sugar, TC, HDL-c, LDL-c and Triglycerides in the general study population. (DOCX 13 kb) [file 12872_2019_1136_MOESM1_ESM.docx]

**Table S1:** Correlation between blood pressure and cardiovascular risk markers (Cases + controls)

| **Parameters** | | **SBP** | **DBP** | **AGE** | **BMI** |
| --- | --- | --- | --- | --- | --- |
| **SBP** | **r**  ***p* value** | 1 |  |  |  |
| **DBP** | **r**  ***p* value** | 0.84^∗∗^  <0.001 | 1 |  |  |
| **AGE** | **r**  ***p* value** | 0.30^∗∗^  0.005 | 0.25^∗^  0.02 | 1 |  |
| **BMI** | **r**  ***p* value** | 0.34^∗∗^  0.001 | 0.40^∗∗^  <0.001 | 0.23^∗^  0.032 | 1 |
| **WC** | **r**  ***p* value** | 0.21^∗^  0.001 | 0.17^∗^  0.008 | -0.05  0.37 | 0.05  0.38 |
| **Blood sugar** | **r**  ***p* value** | 0.47^∗∗^  <0.001 | 0.42^∗∗^  <0.001 | 0.31^∗∗^  0.003 | 0.24^∗^  0.02 |
| **T-C** | **r**  ***p* value** | 0.23^∗^  0.034 | 0.16  0.139 | 0.20  0.055 | 0.31^∗∗^  0.003 |
| **HDL-c** | **r**  ***p* value** | 0.23  0.037 | 0.10  0.335 | -0.006  0.954 | 0.27^∗^  0.010 |
| **LDL-c** | **r**  ***p* value** | 0.15  0.15 | 0.12  0.258 | 0.15  0.162 | 0.30^∗∗^  0.005 |
| **Triglycerides** | **r**  ***p* value** | 0.16  0.128 | 0.18  0.097 | 0.16  0.133 | 0.23^∗^  0.029 |

r, Pearson correlation index; TC, total cholesterol; HDL-c, high density lipoprotein cholesterol; LDL-c, low density lipoprotein cholesterol; WC, waist circumference; **∗∗**, significant correlation with P<0.01; **∗**, significant correlation with p<0.05.
