# Supplementary material for: Positive association between ATP2B1 rs17249754 and essential hypertension: a case-control study in Burkina Faso, West Africa
Source: BMC Cardiovasc Disord. 2019 Jun 26;19:155. doi: 10.1186/s12872-019-1136-x (PMC6595568; doi:10.1186/s12872-019-1136-x)
Supplement: Supplementary file 2 — Table S2. Distribution of systolic and diastolic blood pressure according to genotypes. This file presents the results of association analysis between genetic polymorphisms studied and SBP or DBP in cases and controls. (DOCX 13 kb) [file 12872_2019_1136_MOESM2_ESM.docx]

**Table S2:** Distribution of systolic and diastolic blood pressure according to genotypes

| **SNPs Genotypes** | **Cases (*n*=180)** | |  | **Controls (*n*=200)** | |
| --- | --- | --- | --- | --- | --- |
|  | **SBP (mmHg)** | **DBP (mmHg)** |  | **SBP (mmHg)** | **DBP (mmHg)** |
| **rs2681472 AA** | 167.19 ± 20.23 | 98.63 ± 12.44 |  | 116.50 ± 11.24 | 72.51 ± 8.82 |
| **AG** | 169.11 ± 20.50 | 99.51 ± 9.13 |  | 114.52 ± 13.49 | 72.35 ± 9.37 |
| **GG** | 171.75 ± 6.99 | 97.25 ± 20.90 |  | 114.50 ± 11.44 | 75.50 ± 10.37 |
| ***p* value (AA *versus* AG + GG)** | 0.01∗ | 0.32 |  | 0.001∗ | 0.75 |
| **rs17249754 AA** | 168.00 ± 8.36 | 93.00 ± 13.03 |  | 122.50 ± 11.90 | 78.00 ± 8.90 |
| **AG** | 166.02 ± 17.32 | 98.04 ± 10.95 |  | 111.18 ± 11.16 | 72.06 ± 9.22 |
| **GG** | 168.52 ± 21.93 | 99.43 ± 12.82 |  | 117.39 ± 11.39 | 72.54 ± 8.82 |
| ***p* value (GG *versus* AG + AA)** | < 0.001∗ | 0.001∗ |  | < 0.001∗ | 0.96 |
| **rs3754777 CC** | 167.42 ± 19.52 | 98.86 ± 12.48 |  | 116.25 ± 11.77 | 72.33 ± 8.51 |
| **CT** | 168.04 ± 21.49 | 98.47 ± 11.78 |  | 115.26 ± 11.35 | 72.48 ± 9.95 |
| **TT** | 167.50 ± 25.00 | 97.50 ± 0.50 |  | 122.50 ± 9.57 | 81.25 ± 6.29 |
| ***p* value (CC *versus* CT + TT)** | 0.43 | 0.42 |  | 0.39 | 0.09 |

Values are Median ± SD; Comparison of average SBP and DBP between genotypes (t-test); ∗, significant *p value*; SBP, systolic blood pressure; DBP, diastolic blood pressure; SD, standard deviation.
